# In Vitro Ferrophilic Responses of *Photobacterium damselae* subsp. *piscicida* EKL1 and Characterization of the Fe(III)-Piscibactin Complex

**DOI:** 10.3390/microorganisms13040858

**Published:** 2025-04-09

**Authors:** Asiye Esra Eren Eroğlu, Kadriye Toklu, Nazlı Sarıkahya, İhsan Yaşa

**Affiliations:** 1 Basic and Industrial Microbiology Section, Biology Department, Faculty of Science, Ege University, 35100 Izmir, Türkiye; asiye.esra.eren@ege.edu.tr; 2 Department of Biotechnology, Graduate School of Natural and Applied Sciences, Ege University, 35100 Izmir, Türkiye; kadriye.toklu@gmail.com; 3 Department of Chemistry, Faculty of Science, Ege University, 35100 Izmir, Türkiye; nazli.sarikahya@ege.edu.tr

**Keywords:** deferoxamine, iron homeostasis, NMR, *P. damselae* subsp. *piscicida*, piscibactin, siderophore

## Abstract

The Gram-negative marine bacterium *Photobacterium damselae* subsp. *piscicida* (Pdp) is a pathogen responsible for pseudotuberculosis in various fish species, posing significant threats to aquaculture. Pdp employs strong virulence mechanisms, one of which is the production of the piscibactin siderophore, which plays a key role in iron acquisition from the host. In this study, we evaluated the ferrophilic properties of the Pdp strain EKL1 in relation to growth and biofilm production. In vitro iron limitation significantly suppressed biofilm formation and planktonic growth in EKL1. We then investigated the anti-biofilm activity of deferoxamine (DFO), an iron chelator used to treat transfusion-induced iron overload in thalassemia patients, against EKL1. DFO strongly inhibited EKL1 biofilm production (by 82.1%), suggesting that iron chelation therapy could be an effective strategy to prevent Pdp-induced photobacteriosis outbreaks. Finally, we characterized the iron-bound form of piscibactin through extensive spectroscopic analyses of the siderophore produced by EKL1. Our findings contribute to the development of novel piscibactin-targeted inhibitors, advancing siderophore-based anti-virulence strategies against Pdp.

## 1. Introduction

*Photobacterium damselae* subsp. piscicida (Pdp), a Gram-negative, halophilic bacterium within the Vibrionaceae family, was first isolated in 1963 during mass mortalities among white bass (*Morone americana*) and striped bass (*Morone saxatilis*) populations in Chesapeake Bay, Maryland, USA [1]. Since then, this multiclonal pathogen has been identified across various geographical regions and is recognized as a significant cause of larval mortality, particularly in hatchery environments cultivating economically important aquaculture species, such as *Sparus aurata* (gilthead sea bream) and *Dicentrarchus labrax* (European sea bass) [2,3,4].

Research investigating the pathogenicity of Pdp has demonstrated that water temperature is a critical risk factor influencing virulence, as observed in other members of the Vibrionaceae family [5,6,7]. Several virulence factors have been identified in Pdp, including polysaccharide capsular material involved in host attachment and defense, biofilm production, phospholipases, and extracellular products with cytotoxic and hemolytic activities that cause host tissue damage and facilitate the invasion of neighboring cells [8]. For example, AIP56 exotoxin, a metalloprotease abundantly secreted by virulent strains, has been shown to induce apoptosis in macrophages and neutrophils of European sea bass [9]. It is known that the siderophore (piscibactin) produced by the European clonal strain carrying the pPHDP70 plasmid plays an important role in virulence by enabling iron uptake from the host [10].

Iron (Fe) serves as a critical cofactor for numerous enzymes involved in fundamental biological processes [11]. Dissolved iron (dFe) is an essential micronutrient for microbial growth in marine environments; however, its low solubility and high biological uptake rates result in iron concentrations in surface oceans typically being restricted to picomolar or nanomolar levels [12,13,14]. To overcome these limitations, many members of the Vibrionaceae family, including Pdp, have evolved sophisticated mechanisms for iron acquisition. These include siderophore-mediated pathways and the direct utilization of heme groups from host iron-binding proteins [15].

The biosynthesis and transport of the Pdp siderophore are mediated by a gene cluster known as IRP-HPI, located within the pathogenicity island pPHDP70. The phenolate-catecholate siderophore, known as piscibactin, which is structurally similar to yersiniabactin, was first characterized in 2012, followed by synthetic studies and a detailed analysis of its molecular and chemical structure [15,16,17]. It has been suggested that pathogen virulence is significantly reduced when the irp gene cluster is inactivated or the plasmid is experimentally removed [10].

In this study, we quantitatively evaluated, for the first time, the temperature- and iron availability-dependent growth and biofilm production of the newly isolated Pdp strain EKL1 (OP369288.1) obtained during a pasteurellosis outbreak at an aquaculture facility in Aydın, Turkey. Furthermore, we investigated the antibiofilm efficacy of the iron-chelating agent deferoxamine against EKL1. Finally, we purified the siderophore associated with virulence, produced by the local EKL1 strain, and elucidated its chemical structure using a range of spectroscopic techniques.

## 2. Materials and Methods

### 2.1. Bacterial Strain and Culture Condition

The *Photobacterium damselae* subsp. *piscicida* (Pdp) strain EKL1 (OP369288.1) used in this study was originally isolated from gilthead bream larvae during routine microbiological assessments at the Kılıç Seafood Juvenile Fish Adaptation and Hatchery Facility (Aydın, Türkiye). The culture was lyophilized in skim milk and stored at −20 °C. Before experimental use, cells were retrieved from the frozen stock and reactivated on blood agar, and a single colony was inoculated into tryptic soy broth (TSB, Merck, Darmstadt, Germany) supplemented with 1.5% NaCl and incubated at 25 °C. For experimental preparations, the activated bacteria were diluted in phosphate buffer (pH 7.2) to achieve the appropriate suspension concentrations required for each assay.

### 2.2. Growth and Biofilm Formation Assays

The biofilm assay was conducted using the universal crystal violet method with minor modifications [18]. For the experiments, cells were cultured in an iron-free chemically defined minimal medium (MM9). The MM9 medium was composed of 0.3 g L^−1^ KH_2_PO_4_, 0.5 g L^−1^ NaCl, 1.0 g L^−1^ NH_4_Cl, 6.0 g L^−1^ NaOH, and 30.24 g L^−1^ piperazine-N,N′-bis(2-ethanesulfonic acid) (PIPES). After autoclaving, the medium was supplemented with 30 mL of 10% (*w*/*v*) casamino acids (iron contaminants removed using 3% 8-hydroxyquinoline in chloroform), 2.0 g L^−1^ glucose, 1 mL of 1 M MgCl_2_, and 1 mL of 100 mM CaCl_2_. All supplements were prepared separately, sterilized, and added to the medium under aseptic conditions.

Overnight cultures of Pdp were inoculated into microplate wells containing MM9 supplemented with varying iron concentrations (50 μM, 30 μM, 10 μM, and 0 μM) or LB broth as a control. The plates were incubated at 15 °C and 25 °C for 48 h. Wells containing only the respective media served as negative controls. After incubation, planktonic cells were removed, and the wells were washed twice with PBS. The biofilms were stained with 0.1% crystal violet for 10 min and subsequently rinsed twice with sterile water. Stained biofilms were solubilized with 95% ethanol, and their biomass was quantified by measuring absorbance at OD_590_ nm using a microplate reader (Epoch BioTek, Dover, MA, USA). Each sample was analyzed in triplicate, and mean absorbance values were used for statistical evaluation.

The effect of iron on Pdp growth was assessed using a modified version of previously established protocol [19]. Overnight cultures were inoculated into MM9 broth with and without added iron and incubated at 15 °C and 25 °C. Samples were collected at regular intervals over a period of 0–80 h, and growth was monitored by measuring absorbance at OD_600_ nm. Each experiment was conducted in triplicate, and mean absorbance values were used for analysis. All data were statistically analyzed using GraphPad Prism 8 (San Diego, CA, USA). Results were expressed as mean ± standard deviation, with statistical significance set at *p* < 0.05.

#### Anti-Biofilm Effect of Deferoxamine (DFO)

Commercial deferoxamine (DFO; Desferal, Novartis, Basel, Switzerland) was obtained in powder form. The preparation of the DFO solution was carried out as described in the package insert. Briefly, 500 mg of DFO in each vial was reconstituted in 5 mL of sterile distilled water to achieve a final concentration of 95 mg/mL.

The in vitro antibiofilm activity of DFO against Pdp strain EKL1 and the test organism *V. anguillarum* Gdp19 (PP270135) biofilms was assessed using a spectrophotometric microplate assay [18]. Briefly, EKL1, at a concentration of 1.0 × 10^7^ CFU/mL, was treated with 2.5, 5, 7.5, and 10 µg/mL DFO and incubated in a 96 well plate for 48 h. Wells containing EKL1 and Gdp19 cultures without DFO were used as controls. After the incubation period, the well contents were aspirated, and the microplates were subjected to crystal violet (CV) staining as described above. Spectrophotometric measurements were performed to obtain OD values. The percentage of biofilm degradation was calculated using the following formula: Biofilm degradation (%) = (OD_A_ − OD_B_)/OD_A_ × 100, where OD_A_ represents the OD of the biofilm control well without DFO and OD_B_ represents the OD in the presence of DFO.

### 2.3. Evaluation of Siderophore Production

The siderophore production process required the preparation of a growth medium with sufficient iron deficiency. To achieve this, all glassware was acid-washed overnight in 6 M HCl, thoroughly rinsed with ultrapure water, and sterilized by autoclaving prior to use [20]. Siderophore production by Pdp was subsequently evaluated using both qualitative and quantitative methods.

#### 2.3.1. Qualitative Assay

The Chrome Azurol S (CAS) agar plate method, as described by Schwyn and Neilands (1987), was employed to qualitatively assess siderophore production [19]. Briefly, Pdp was cultured in MM9 medium on a shaker at 120 rpm and 25 °C for 24 h. Subsequently, 6 µL of a bacterial suspension containing 1 × 10^8^ cells/mL was spotted onto CAS agar plates and incubated at 25 °C for 5 days. At the end of the incubation period, the presence of an orange-yellow halo surrounding the colonies on the CAS blue agar indicated siderophore production.

#### 2.3.2. Quantitative Assay

To quantitatively assess siderophore production, Pdp was pre-incubated overnight in iron-limited MM9 liquid medium. Subsequently, 100 µL of the culture was inoculated into freshly prepared MM9 medium. The cultures were incubated at 25 °C for 80 h in a shaking incubator at 120 rpm. Samples were collected at 12, 24, 36, 48, 72, and 80 h during the incubation period. Each sample was centrifuged at 8000 rpm for 15 min at 20 °C to separate the supernatant. A 0.5 mL aliquot of the supernatant was transferred into a sterile tube and mixed with an equal volume of freshly prepared CAS solution. To enhance binding between the CAS solution and siderophore molecules and to intensify the resulting color change, 10 µM of shuttle solution was added. After 15–20 min, a color change from blue to red, orange, or yellow indicated positive siderophore production. Sterile MM9 medium served as the negative control, while MM9 medium supplemented with commercial DFO acted as the positive control. For quantification, standard concentrations of DFO, the negative control, and Pdp supernatants containing siderophores were treated with an equal volume of CAS reagent. The optical density was measured at 630 nm, and siderophore units (%SU) were calculated using the formula: % of siderophore units = Ar − AS /Ar ×100, where Ar = absorbance of reference at 630 nm and As = absorbance of sample at 630 nm.

### 2.4. Production and Characterization of Piscibactin

Pdp strain EKL1 was incubated at 25 °C for 72 h in a 250 mL Erlenmeyer flask containing 100 mL of MM9 media. Following incubation, the bacterial cells were pelleted by centrifugation at 8000 rpm for 20 min. The resulting supernatant was subjected to the CAS assay to confirm the presence of siderophores.

The supernatants were sterilized sequentially by filtration through 0.45 µm and 0.22 µm pore-sized filters. To eliminate inorganic salts and other medium components, an extraction process was performed on the filtrates with 1:1 butanol:water and 1:1 chloroform:water mixture, respectively. Purification of the extracted samples was carried out using reverse-phase chromatography on an RP-18 silica column. Chromatography was performed under conditions protected from light and temperature, starting with 100% water as the solvent system, followed by an increase in the percentage of methanol in each step [21]. The collected fractions were further analyzed using silica gel thin-layer chromatography (TLC) by the CHCl_3_:MeOH:H_2_O 61:32:7 solvent system, which revealed three main fractions. The fraction containing the siderophore molecule was re-evaluated on a normal-phase silica TLC plate by the CHCl_3_:MeOH:H_2_O 61:32:7 solvent system and visualized under UV light at 254 nm.

#### Nuclear Magnetic Resonance (NMR) Spectroscopy

The structure of the purified siderophore compound was initially characterized using ultraviolet (UV) and Fourier transform infrared (FT-IR) spectroscopy to identify the functional groups present in the molecule. Subsequently, one-dimensional ^1^H and ^13^C NMR (nuclear magnetic resonance) spectra were recorded to determine the number and types of carbon and hydrogen atoms in the compound.

To achieve a comprehensive structural elucidation, advanced one-dimensional techniques, including Distortionless Enhancement by Polarization Transfer (DEPT), and two-dimensional NMR techniques, such as Heteronuclear Multiple Bond Correlation (HMBC), Heteronuclear Single Quantum Coherence (HSQC), Correlation Spectroscopy (COSY), and Nuclear Overhauser Effect Spectroscopy (NOESY), were employed [22,23]. DEPT analysis allowed the identification of methyl, methylene, and methine groups, while HSQC revealed ^1^H-^13^C correlations, mapping specific protons to their respective carbons. COSY provided information on neighboring proton interactions through ^1^H-^1^H couplings, and NOESY presented insights into the compound’s three-dimensional conformation. HMBC analysis confirmed two- and three-bond couplings, establishing intramolecular linkages and overall structural connectivity.

Finally, high-resolution electrospray ionization mass spectrometry (HRESIMS) was conducted to determine the molecular formula and molecular weight of the compound. Fragmentation patterns obtained from the mass spectrometry analysis further supported the structural identity of the siderophore.

## 3. Results

### 3.1. The Role of Ferric Iron in the Biofilm Production and Growth

**Figure 1:**
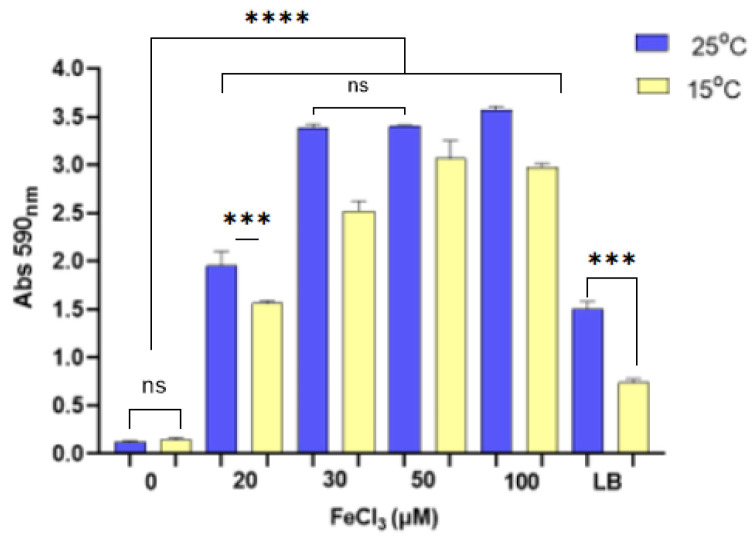
Biofilm production of Pdp EKL-1 incubated at different temperatures in minimal media supplemented with varying concentrations of FeCl_3_ (20, 30, 50, and 100 µM) and without FeCl_3_ supplementation, as well as in LB broth. The abbreviation “ns” indicates that the *p*-value is greater than 0.05 and not statistically significant (*p* > 0.05). Three asterisks (***) indicate a *p*-value less than 0.05 (*p* < 0.05). Four asterisks (****) indicate a *p*-value less than 0.001 (*p* < 0.001).

The effect of iron availability and concentration on biofilm production, a critical determinant of Pdp virulence, was evaluated. For this purpose, Pdp cells were cultured in microplate wells containing either iron-free or iron-supplemented MM9 medium, as well as LB broth. The results indicated that iron deficiency significantly inhibited biofilm formation. The highest level of biofilm production was observed when Pdp was cultured at 25 °C in MM9 medium supplemented with 100 μM FeCl_3_. At 25 °C, the optical density (OD) of biofilms in MM9 medium supplemented with 30 μM and 50 μM FeCl_3_ was recorded as 3.382 and 3.403, respectively, with no statistically significant difference between these values (Figure 1). In contrast, biofilm formation in medium containing 20 μM FeCl_3_ was significantly lower, with an OD of 1.948 (ANOVA, *p* < 0.05). Additionally, cells grown in iron-supplemented MM9 media produced significantly more biofilm compared to those cultured in conventional LB broth. Notably, biofilm production in iron-free MM9 medium was reduced by over 93.99% compared to iron-supplemented conditions.

**Figure 2:**
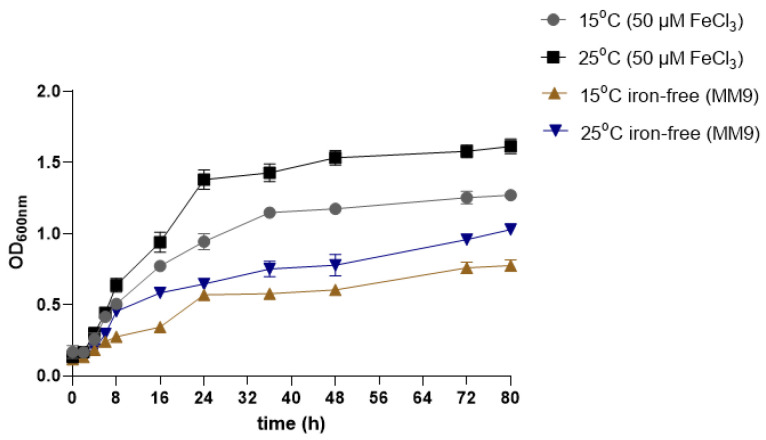
Time-dependent optical density at 600 nm of Pdp cells incubated at different temperatures in iron-supplemented and non-supplemented MM9 media. The data presented herein represent the mean values of three independent experiments. Error bars represent standard deviations. Yellow and blue arrows indicate iron-free media, while black and grey arrows indicate iron-supplemented media.

The OD of Pdp cultures was monitored at regular intervals over 80 h under both iron-supplemented and iron-deficient conditions at 15 °C and 25 °C. At both temperatures, Pdp exhibited a prolonged lag phase of approximately 8 h in the iron-supplemented medium, whereas the lag phase extended to approximately 10 h in the iron-deficient medium. Following the lag phase, a rapid increase in OD was observed under iron-supplemented conditions, while iron limitation significantly restricted the growth of planktonic cells. By the end of the experiment, the highest OD (1.614) was recorded at 25 °C in the presence of iron. Cultures grown at 15 °C under iron-supplemented conditions reached an OD of 1.271. In contrast, under iron-deficient conditions, the ODs were significantly lower, measuring 1.029 at 25 °C and 0.776 at 15 °C (Figure 2).

### 3.2. Anti-Biofilm Effect of Deferoxamine Mesylate (DFO)

**Figure 3:**
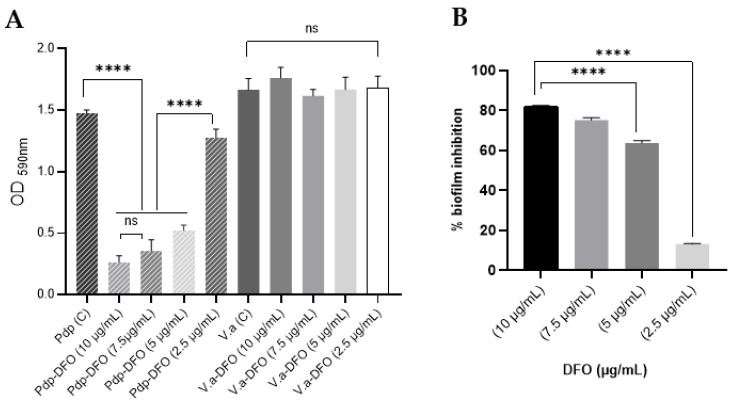
Inhibitory effect of DFO on biofilm formation. (**A**) Biofilm production capacity of Pdp strain EKL1 and *V.anguillarum* strain GDP19 measured at 590 nm wavelength in the presence and absence of DFO. (**B**) Percentage of biofilm inhibition rates of EKL1 in the presence of DFO. The abbreviation “ns” indicates that the *p*-value is greater than 0.05 and not statistically significant (*p* > 0.05). Four asterisks (****) indicate a *p*-value less than 0.001 (*p* < 0.001).

In order to investigate the antibiofilm effects of DFO against Pdp, *V. anguillarum* strain Gdp19, which was previously isolated in our studies, was used as a test organism for comparison. DFO exhibited markedly different effects among the tested organisms (Figure 3A), demonstrating a strong inhibitory effect on biofilm production by Pdp EKL1. The addition of 2.5, 5, 7.5, and 10 µg/mL DFO to the growth medium resulted in a 13.3%, 64.6%, 75.9%, and 82.1% inhibition of EKL1 biofilm production, respectively (Figure 3B). However, this effect was not observed in Gdp19; on the contrary, the addition of 10 µg/mL DFO to the growth medium resulted in a 5.7% increase in biofilm production.

### 3.3. Siderophore Production

**Figure 4:**
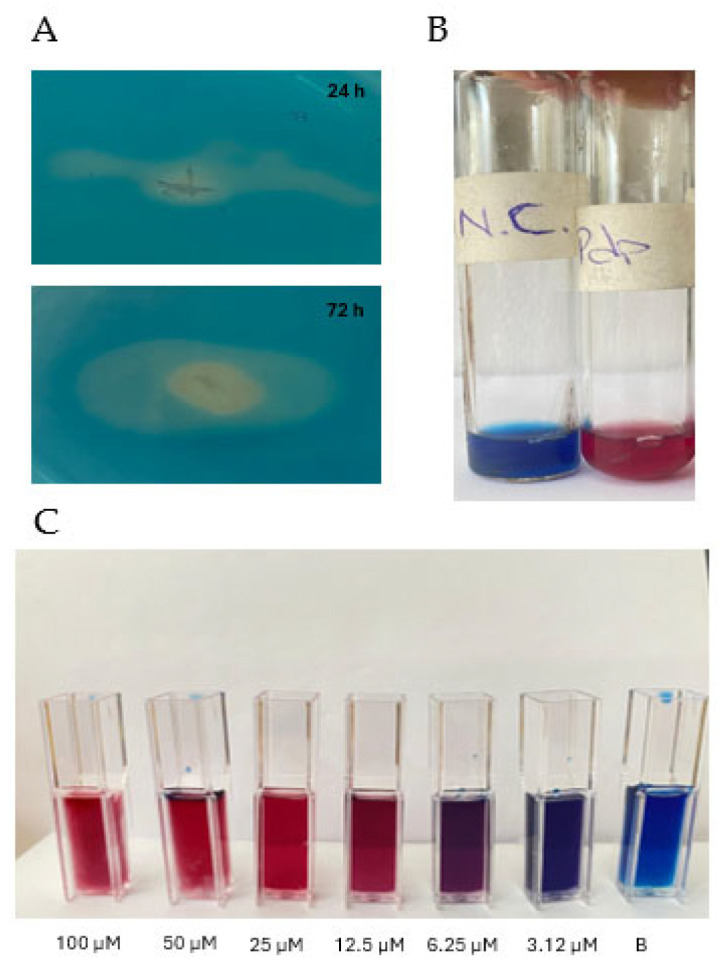
Results of the CAS agar (**A**) and CAS broth (**B**) assays for Pdp incubated at different temperatures. Standard dilution (**C**) generated using decreasing concentrations of DFO solution with CAS dye (where “B” refers to the control, containing only CAS dye and sterile PBS).

**Figure 5:**
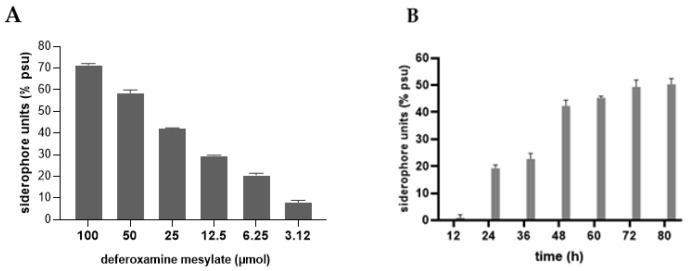
Percentage of siderophore units (% PSU) corresponding to DFO standards (**A**). Time-dependent siderophore production by Pdp cells (**B**). Data presented represent mean values of three independent experiments. Error bars represent standard deviations.

When cultured on CAS agar, Pdp colonies produced a yellow halo surrounding the bacterial growth, indicating the production of an Fe-binding compound (Figure 4A). To quantify siderophore production, a calibration curve was established using DFO standards to determine the corresponding PSU levels (Figure 4B and Figure 5A). The Pdp isolate was then incubated in iron-limited MM9 liquid medium for 80 h at 25 °C (Figure 4B). Siderophore production began at 24 h and reached a maximum of 50.3 PSU at 80 h, corresponding to approximately 70 µM siderophore (Figure 5A,B).

### 3.4. Purification of Pdp Siderophore

Based on the results of thin-layer chromatography (TLC), fractions collected from the RP column were separated into individual tubes (Figure S1). According to subsequent silica gel TLC analyses (solvent system CHCl_3_:MeOH:H_2_O 61:32:7), a total of six different fractions were identified. A total of 99.7 mg of metal-bound siderophore from two fractions and 70.4 mg free siderophore from one fraction were successfully isolated and purified. Prior to structural characterization studies, the purified samples were stored at −20 °C.

### 3.5. Determination of the Structure of Siderophore by Spectroscopic Techniques

**Figure 6:**
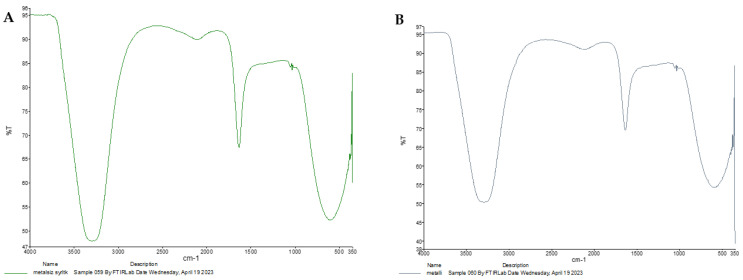
FT-IR spectra of free piscibactin (**A**) and Fe(III)-bound piscibactin (**B**).

The structure of the purified siderophore was identified as consisting of two forms: one as an Fe-bound piscibactin complex and the other as a free piscibactin molecule. To analyze the functional groups, double bonds, and interactions within the isolated compounds, FT-IR spectroscopy was employed. A sharp peak observed around 1650 cm^−1^ clearly indicated the presence of carboxylic acid groups. Peaks within the 3750–3300 cm^−1^ range corresponded to O-H and N-H bonds, while peaks observed at 1675–1500 cm^−1^ represented C=C and N=C double bonds. The region between 1250–600 cm^−1^, known as the fingerprint region, showed C-H interactions. The observed peaks were consistent for both the Fe-complexed and free siderophore forms, with minor differences in the fingerprint region being characteristic of the Fe complex (Figure 6A,B). To further elucidate these distinctions, UV spectroscopic analysis was performed.

**Figure 7:**
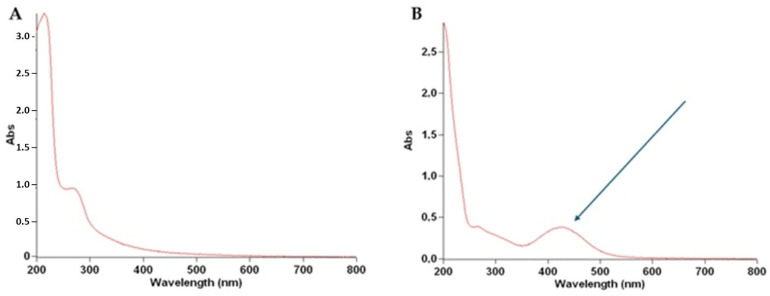
UV spectra of free piscibactin (**A**) and Fe(III)-bound piscibactin (**B**).

Comparison of the UV spectra of the purified siderophores (Figure 7A,B) revealed a distinct absorbance peak between 400–500 nm in the Fe-bound siderophore complex, as expected. This absorbance corresponds to the d→d* and t_2g_→e_g_ electronic transitions typical of Fe complexes. Unlike the FT-IR spectra, which were less effective in distinguishing between the free and Fe-bound forms, the UV analysis clearly confirmed the presence of the Fe complex. The UV absorbance values of the purified siderophores are presented in Table S1.

**Figure 8:**
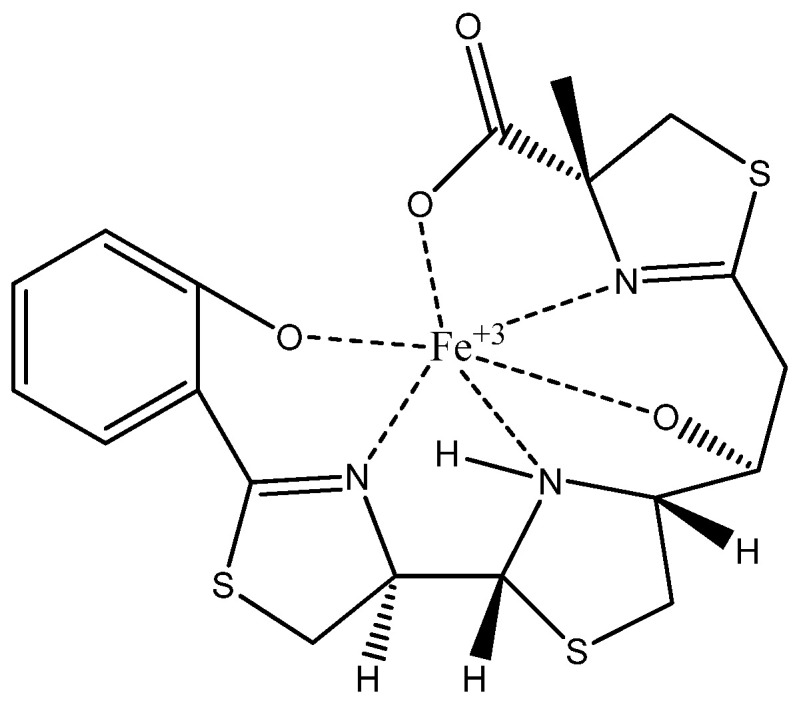
Fe(III)-piscibactin complex structure.

The spectra shown in Supplementary Figures S2–S10 depict the one-dimensional and two-dimensional NMR analyses performed to determine the precise structure of the siderophores in both free and Fe-bound forms. These spectra enabled the identification of specific carbon and proton positions, as well as connection points within the structures. Based on these analyses, the siderophores were definitively identified as piscibactin in both free and Fe-bound forms, as illustrated in Figure 8, the six-coordinate Fe-bound piscibactin complex structure (Tables S2 and S3).

**Figure 9:**
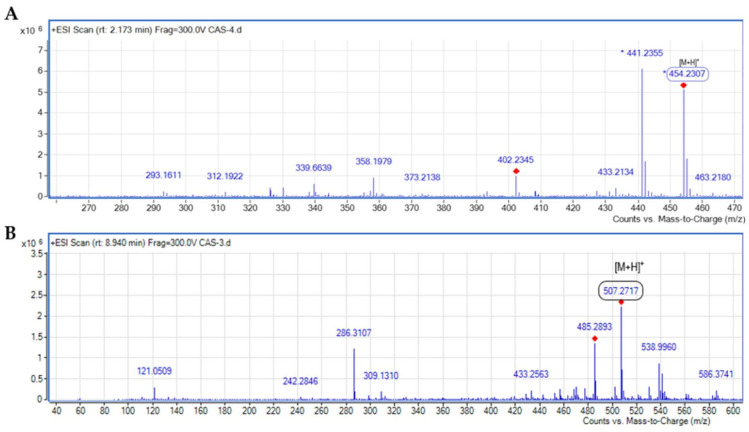
Mass spectra of (**A**) piscibactin and (**B**) Fe(III)-bound piscibactin complexes.

The mass spectra of both the free and Fe-bound siderophores were obtained using high-resolution electrospray ionization mass spectrometry (HR-ESIMS), with molecular masses determined to four decimal places (Figure 9). The molecular mass of free piscibactin was identified as *m*/*z*: 454.2307 [M+H]^+^, while the Fe-piscibactin complex exhibited a mass of *m*/*z*: 507.2717 [M+H]^+^. These final mass spectrometry analyses confirmed the molecular structures of the purified siderophores with high precision.

## 4. Discussion

In aquaculture environments, the increasing frequency of disease outbreaks driven by global warming, combined with the limitations of existing chemotherapeutic antibiotics, has prompted researchers to explore alternative, non-antibiotic therapeutic strategies [24,25]. In this context, there has been a growing emphasis on studying the virulence factors of pathogens and their interactions with environmental factors. Such research provides a crucial foundation for the development of pathogen-specific treatment approaches [26,27]. Pathogenic members of the Vibrionaceae family, in particular, have emerged as ideal model organisms for understanding how pathogenicity evolves from environmental ancestors and how genetic diversification mechanisms shape bacterial populations [24].

*Photobacterium damselae* subsp. *piscicida* (Pdp), a member of the Vibrionaceae family, is noteworthy for its broad geographic distribution, lack of host specificity, and association with high mortality rates. It is the causative agent of one of the most lethal bacterial diseases in aquaculture, known as pasteurellosis. Since the 1990s, Pdp has been reported as a major pathogen responsible for outbreaks, particularly in gilthead seabream (*Sparus au-rata*) and European sea bass (*Dicentrarchus labrax*) farming in Türkiye and other Mediterranean countries. Among the virulence mechanisms of Pdp, the piscibactin-mediated iron acquisition system has been identified as a key factor in its pathogenicity in fishery [28,29].

We investigated the effect of iron availability on biofilm production, a critical determinant of Pdp virulence. Our results demonstrated that iron deficiency significantly inhibited biofilm formation in Pdp. The highest biofilm production was observed when Pdp was cultured in MM9 medium supplemented with 100 μM FeCl_3_ at 25 °C. Although biofilm production was lower at 15 °C than 25 °C, increasing iron concentrations enhanced biofilm formation at both temperatures. The crucial role of iron in biofilm development was first highlighted by the discovery that lactoferrin, a high-affinity Fe^3+^-binding protein, inhibits biofilm formation in *Pseudomonas aeruginosa* [30]. The present study revealed that high iron levels triggered *P. aeruginosa* to form microcolonies that later developed into mature biofilms, whereas low iron levels suppressed biofilm growth. Similarly, Gentile et al. (2014) reported that *Acinetobacter baumannii* strains from veterinary and clinical sources exhibited diverse biofilm-associated phenotypes in response to iron restriction [31]. Among these isolates, 42% showed a significant increase in biofilm production, 24% exhibited reduced biofilm growth, and the remaining 34% were unaffected by iron availability. These findings further emphasize the complex and variable role of iron in regulating biofilm dynamics among different bacterial species.

The vegetative cell growth of Pdp was monitored at regular intervals over 80 h under both iron-supplemented and iron-limited conditions at 15 °C and 25 °C. At both temperatures, optical density increased rapidly under iron-supplemented conditions, whereas iron limitation significantly restricted the growth of planktonic cells. Previous studies have reported that, despite the broad tolerance of Vibrionaceae members to temperature and salinity variations, iron availability is a key factor in their growth, particularly in the absence of thermal or osmotic stress [32]. In many regions of the surface ocean where iron is scarce, competition for iron sources plays a crucial role in shaping microbial community structure [33]. For instance, the marine bacterium *V. fischeri* ES114 enhances its iron acquisition and reproductive ability by producing and secreting the siderophore aerobactin while simultaneously inhibiting the growth of competing species by limiting iron availability [34]. Siderophore biology provides insight into the complex functional dynamics of microbial communities, influencing competition, synergism, and altruism, processes that are partially regulated by siderophore production and uptake [35].

Having evaluated the in vitro ferrophilic properties of Pdp EKL1, we explored the potential usefulness of iron chelation therapy in combating it. To this end, we investigated the anti-biofilm activity of deferoxamine (DFO), a semi-synthetic iron chelator approved for medical use. Iron chelators such as DFO have previously been studied as potential treatments for multidrug-resistant Gram-negative bacterial infections [36,37]. In our study, the *V. anguillarum* Gdp19 strain was used as a test organism for comparison. The results demonstrated that DFO exhibits anti-biofilm activity against Pdp EKL1, even at very low concentrations. In contrast, Gdp19 was not affected by DFO; rather, the presence of DFO appeared to induce biofilm formation in this strain. This observation suggests that while Gdp19 may utilize DFO as an iron source, EKL1 is unable to do so and consequently loses in the competition for iron acquisition from the medium. The inhibitory effect of DFO is likely due to its strong iron-binding capacity. It has been reported that DFO, a hydroxamine-type siderophore produced by *Streptomyces* species, can be utilized by certain bacteria, such as *V. vulnificus* and *S. aureus*, for efficient iron uptake via specific receptors [37,38]. The promising aspect of iron chelators is that those not recognized or utilized by bacterial cells can significantly suppress the growth of ferrophilic pathogens. In a previous study, pretreatment of juvenile rainbow trout with *P. fluorescens* AH12, a siderophore producer, resulted in a 46% reduction in mortality following challenge with *V. anguillarum* [39,40]. Similarly, another study reported that pyoverdine secreted by *P. aeruginosa* PA1 may serve as a promising agent for vibriosis treatment [41]. In this context, our findings suggest that iron chelation therapy may serve as an effective strategy to prevent Pdp-induced photobacteriosis outbreaks by restricting iron availability.

For the quantitative determination of siderophore production in Pdp EKL1, deferoxamine (DFO) in its mesylate form was used as a standard. Pdp cells produced 50.3 PSU (approximately 70 µM) of siderophores within 72 h at 25 °C. The expression and broad distribution of piscibactin have also been observed in other Vibrionaceae members, particularly at lower temperatures. This widespread distribution has been attributed to evolutionary mechanisms, likely driven by horizontal gene transfer (HGT), as an adaptive strategy to facilitate host infection in colder environments [42,43].

The structure of the phenolate-catecholate siderophore piscibactin, produced by the European Pdp strain, has been well characterized since 2012 [15,16,17]. However, in this study, we aimed to conduct a detailed structural comparison of the siderophore produced by this newly isolated pathogenic strain and to obtain the iron-bound form of piscibactin for further investigations. Therefore, advanced spectroscopic techniques, such as FT-IR, UV, 1D, 2D-NMR, and mass analysis, were performed on both free and iron-bound siderophores to determine the exact structures. The resulting spectra provided detailed information on the precise number of the carbon and proton atoms, their connections, and the overall molecular structure. These analyses confirmed that the siderophore structure is piscibactin, both in its free and iron-bound forms. Additionally, the six-coordinate Fe(III)-bound complex structure of piscibactin was identified. High-resolution HR-ESI-MS mass spectrometry was employed to determine the molecular masses of the free and Fe-bound piscibactin. The mass of free piscibactin was found to be *m*/*z*: 454.2307 [M+H]^+^, while the Fe(III)-piscibactin complex had a mass of *m*/*z*: 507.2717 [M+H]^+^. These values align with the mass spectrometry data reported in the literature, and the fragmentation patterns are consistent with the identified structure. Additionally, the fragmentation patterns observed in the mass spectra align with the proposed structures. These findings have confirmed the molecular structure of free piscibactin and Fe(III)-piscibactin complex. A comparison was also discussed between the NMR values of the Fe(III)-piscibactin complex and the Ga(III)-complex in the literature. There are some modifications due to NMR analyzes of both the different metals and solvents. The difference gradually increased and reached the expected level. Similarly, since there is no literature on the NMR values of pure piscibactin, a comparison was made with the NMR values of pure yersiniabactin, and it was found that the one- and two-dimensional NMR analysis values of pure piscibactin were compatible [15,44]. As a result, the piscibactin siderophore isolated from the Pdp strain was found to be highly homologous to the phenolate-catecholate-type yersiniabactin system in its structure. However, piscibactin can be distinguished from yersiniabactin by the absence of two methyl groups at the C14 position. This difference may be explained by the fact that Photobacterium’s PKS/NRPS Irp1 enzyme counterpart, HMWP1, has only one MT domain instead of the two MT domains found in yersiniabactin [45,46].

Recent advances in molecular simulation-based studies have significantly accelerated the discovery of novel antimicrobial and antivirulence strategies by enabling the rapid screening and evaluation of a vast number of molecules [47]. In this study, we successfully obtained the iron-bound piscibactin complex structure, providing a foundation for future modeling, simulation, and docking studies aimed at its inhibition. This is particularly crucial because siderophores exert their biological function primarily by forming stable complexes with iron (Fe^3+^). In its unbound state, a siderophore serves merely as a precursor molecule, whereas its biologically active form is the iron-complexed structure. The binding of iron alters the electronic configuration, molecular conformation, and binding kinetics of the siderophore, significantly influencing its interactions with bacterial receptors and host defense mechanisms [48]. By establishing a well-defined model of the iron-bound structure, this study provides a foundation for more accurate computational simulations that can better reflect real biological conditions. We anticipate that these results will facilitate the development of targeted inhibitors and novel therapeutic interventions, contributing to the advancement of siderophore-based anti-virulence strategies in bacterial pathogenesis.

## 5. Conclusions

Within the framework of One Health, therapeutic anti-virulence strategies in aquaculture have emerged as a promising alternative to traditional biocidal approaches for combating infectious diseases. In our study, we evaluated, for the first time, the effect of an iron chelator against Pdp. Our findings demonstrate that DFO significantly inhibits Pdp biofilm production. While the potential application of DFO for fish treatment depends on factors such as fish species and disease severity, possible administration methods include incorporation into culture water, addition to feed for systemic absorption, or direct injection for acute cases. However, the stability and practical feasibility of each method require further investigation to determine the most effective approach for aquaculture use. Overall, our findings indicate that piscibactin represents a promising therapeutic target for mitigating Pdp infections. Such targeted strategies offer a more refined approach to pathogen control, prioritizing aquatic animal health while promoting environmental sustainability.

## Data Availability

The raw data supporting the conclusions of this article will be made available by the authors on request.

## Supplementary Materials

The following supporting information can be downloaded at: https://www.mdpi.com/article/10.3390/microorganisms13040858/s1, Table S1: The UV absorbance values of the purified siderophores; Table S2: The following comparison is made of the nuclear magnetic resonance (NMR) values of isolated and purified piscibactin-Fe+3 and the literature piscibactin-Ga+3 complexes; Table S3: NMR values for the isolated and purified piscibactin and literature-reported yersiniabactin; Figure S1: TLC fractions obtained during the purification of piscibactin; Figure S2: 1H-NMR spectra of piscibactin (400 MHz, D_2_O); Figure S3: 1H-NMR spectra of Fe(III)-bound piscibactin (400 MHz, D_2_O); Figure S4: 13C-NMR spectra of piscibactin (100 MHz, D_2_O); Figure S5: 13C-NMR spectra of Fe(III)-bound piscibactin (100 MHz, D_2_O); Figure S6: COSY and HSQC spectra for piscibactin; Figure S7: COSY and HSQC spectra for Fe(III)-bound piscibactin complexes; Figure S8: HMBC and DEPT spectra for Fe(III)-bound piscibactin complexes; Figure S9: NOESY spectra for piscibactin; Figure S10: HMBC spectra for piscibactin.
