# Supplementary material for: In Vitro Ferrophilic Responses of Photobacterium damselae subsp. piscicida EKL1 and Characterization of the Fe(III)-Piscibactin Complex
Source: Microorganisms. 2025 Apr 9;13(4):858. doi: 10.3390/microorganisms13040858 (PMC12029771; doi:10.3390/microorganisms13040858)
Supplement: Supplementary file 1 [file microorganisms-13-00858-s001.zip › microorganisms-3538991 Figures.pdf]

Supplementary figures

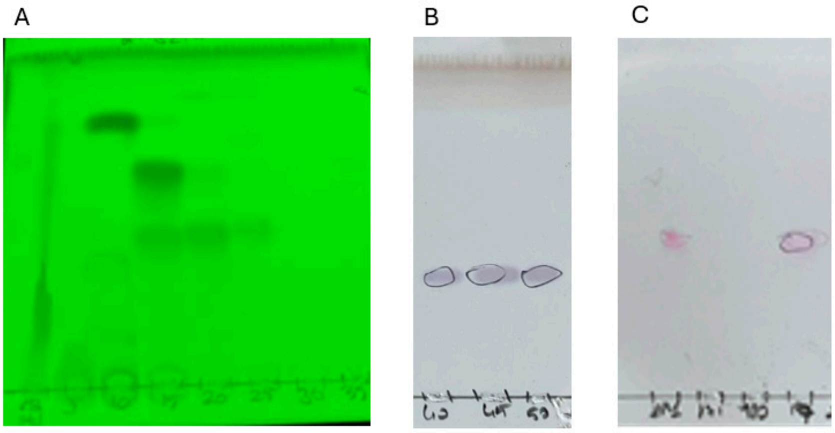

Figure S1: TLC fractions obtained during the purification of piscibactin. (A) Fractions collected from the RP column visualized under 254 nm UV light. Metal-free siderophore (70.4 mg) (B) and Fe-bound siderophore (56.7 mg) (C) were observed using silica TLC with a solvent system of 61:32:7 $CHCl_3$:MeOH:H_2O.

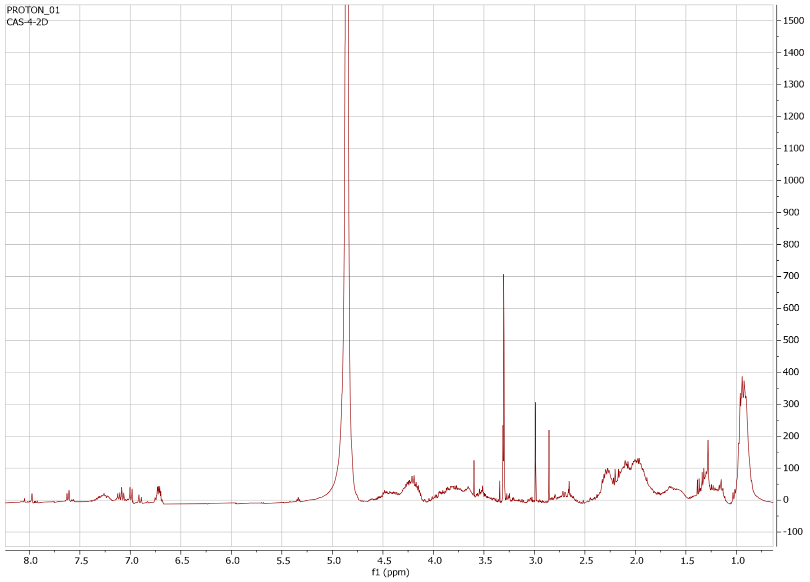

Figure S2: 1H-NMR spectra of piscibactin(400 MHz, D_2O).

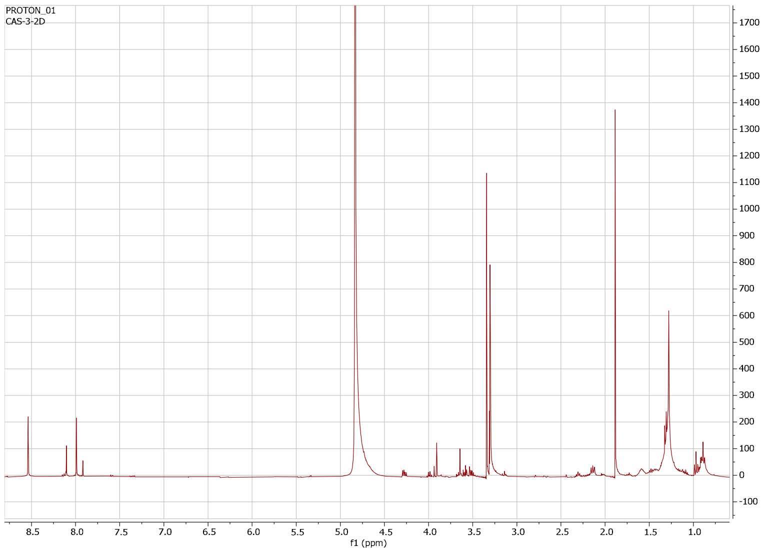

Figure S3: 1H-NMR spectra of Fe(III)-bound piscibactin (400 MHz, D_2O).

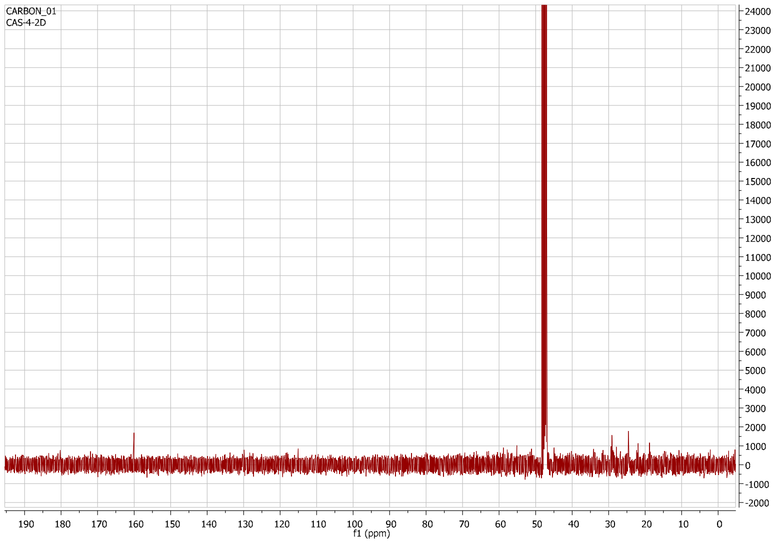

Figure S4:13C-NMR spectra of piscibactin (100 MHz, D_2O).

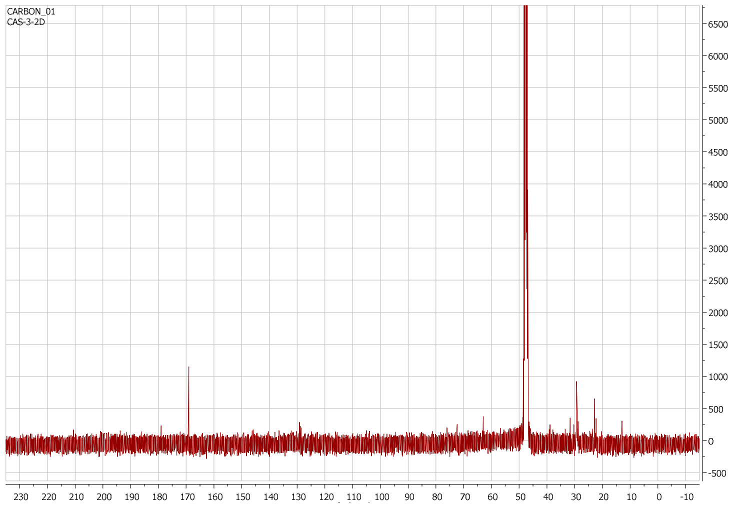

Figure S5: 13C-NMR spectra of Fe(III)-bound piscibactin (100 MHz, D_2O).

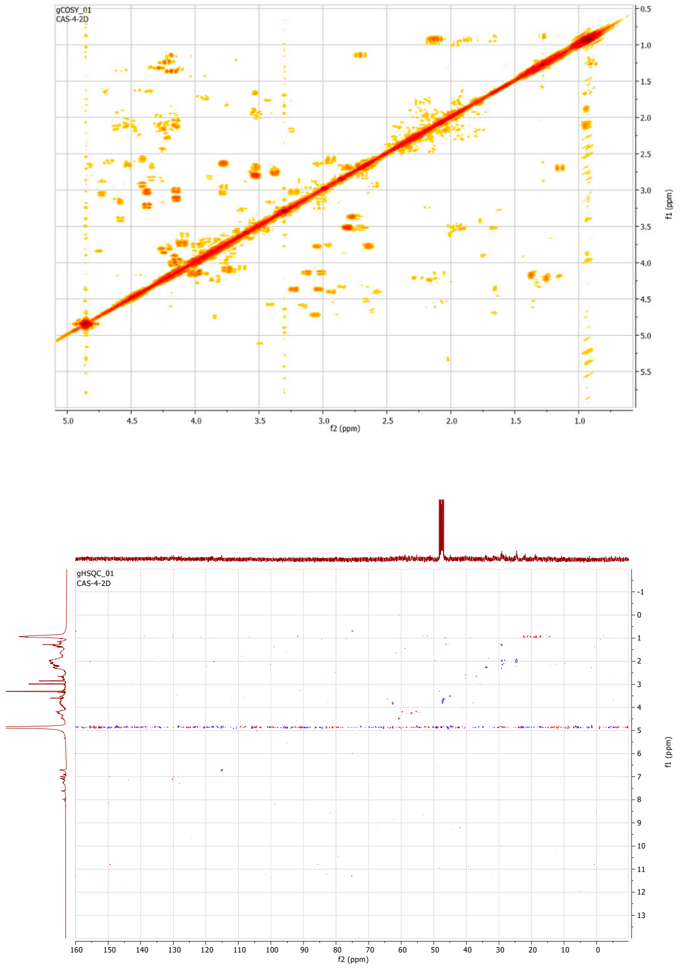

Figure S6: COSY and HSQC spectra for piscibactin

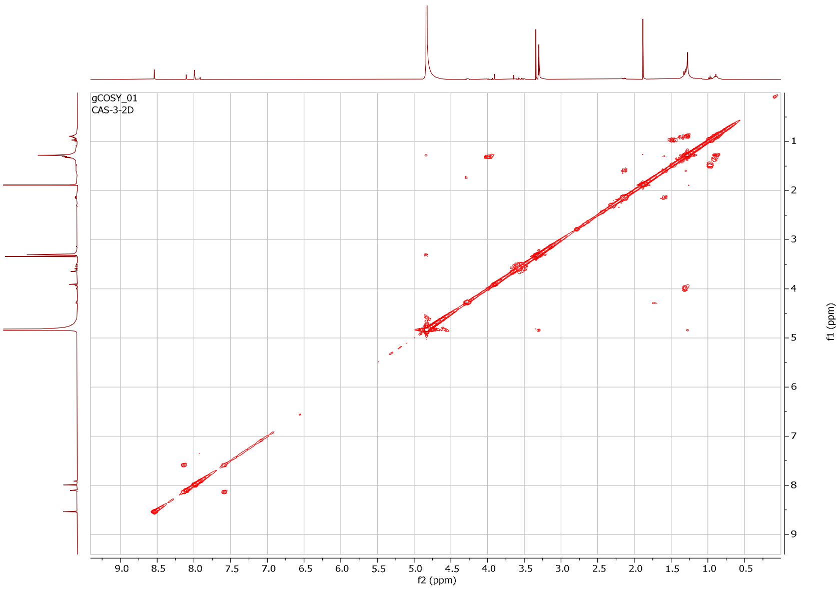

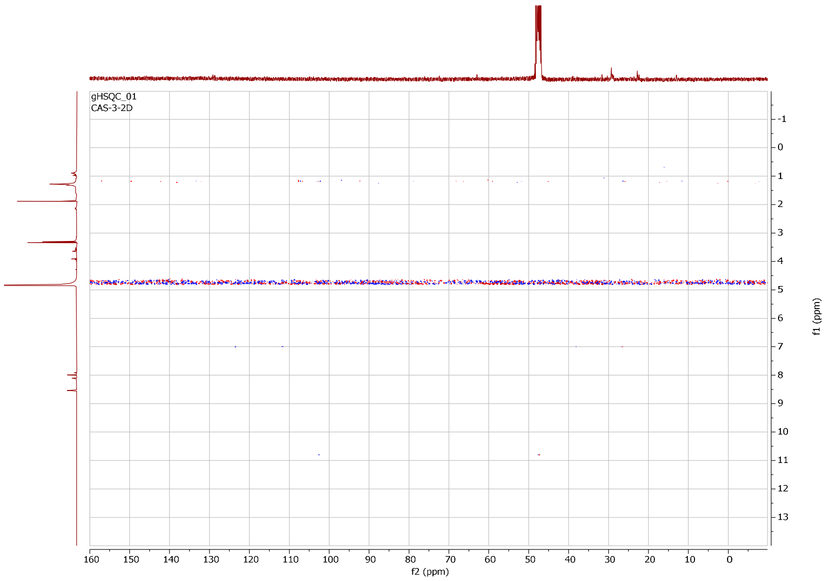

Figure S7: COSY and HSQC spectra for Fe(III)-bound piscibactin complexes.

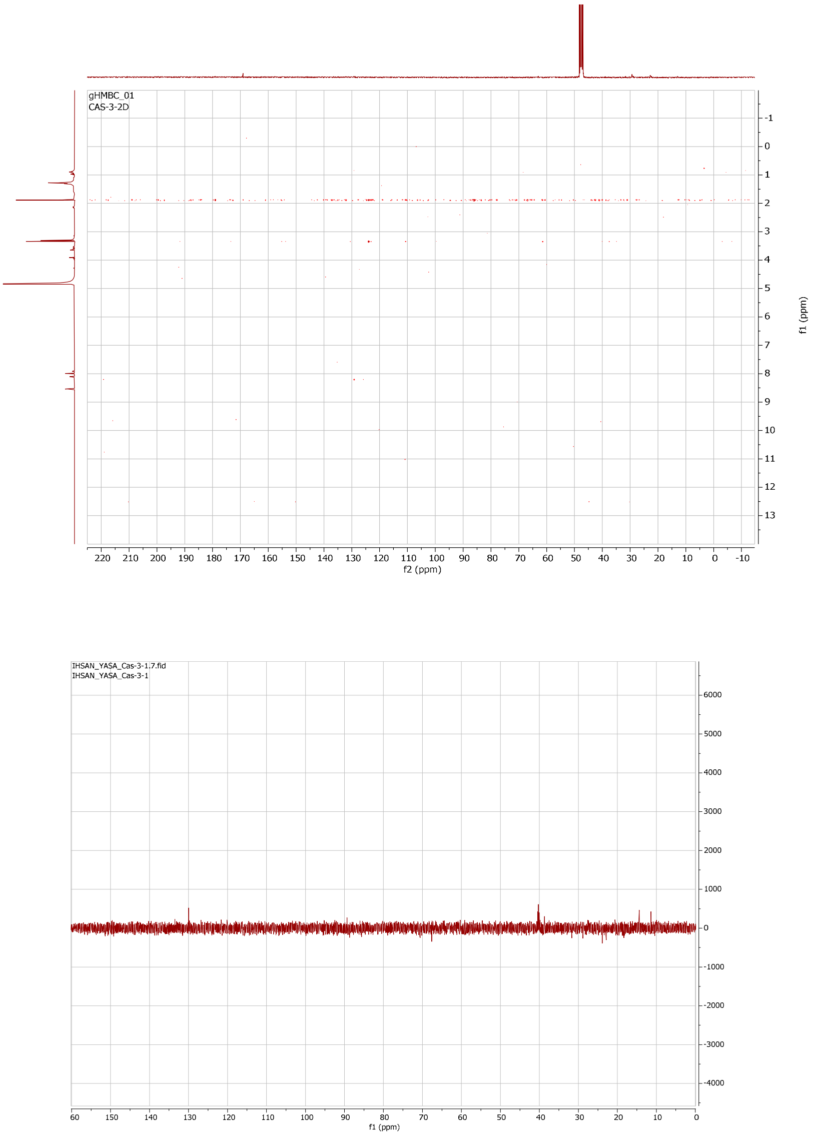

Figure S8:HMBC and DEPT spectra for Fe(III)-bound piscibactin complexes

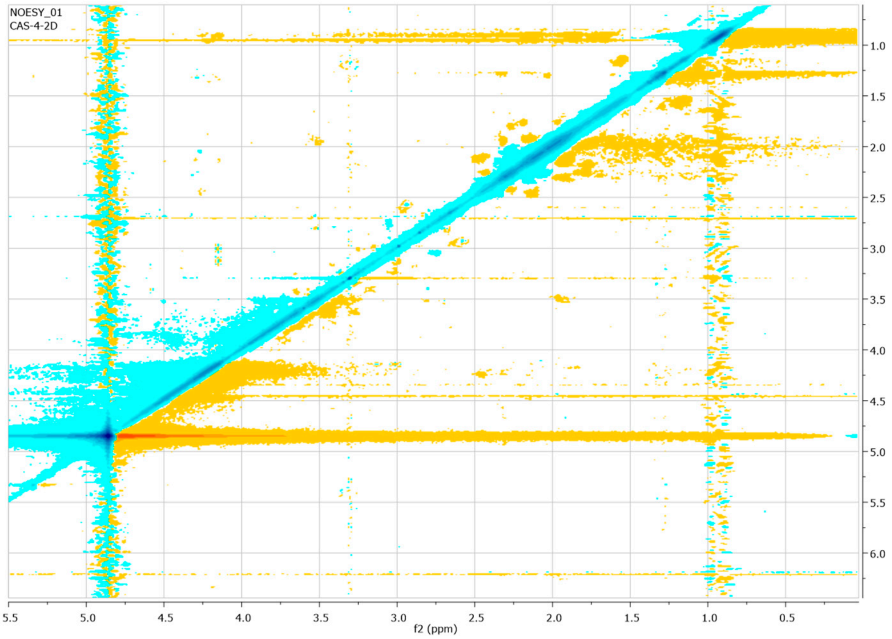

Figure S9: NOESY spectra for piscibactin

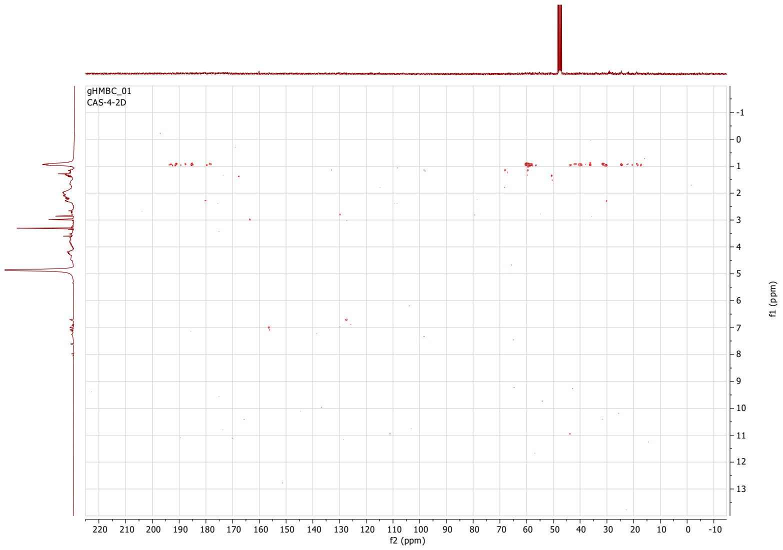

Figure S10: HMBC spectra for piscibactin
