# Supplementary material for: In Vitro Ferrophilic Responses of Photobacterium damselae subsp. piscicida EKL1 and Characterization of the Fe(III)-Piscibactin Complex
Source: Microorganisms. 2025 Apr 9;13(4):858. doi: 10.3390/microorganisms13040858 (PMC12029771; doi:10.3390/microorganisms13040858)
Supplement: Supplementary file 1 [file microorganisms-13-00858-s001.zip › microorganisms-3538991 Tables.pdf]

Supplementary Tables

Supplementary Table S1: The UV absorbance values of the purified siderophores.

Piscibactin UV values		Piscibactin-Fe^{III} bound UV values	
**Wavelength (nm)**	**Abs**	**Wavelength (nm)**	**Abs**
787,00	-0,001	689,00	0,011
732,00	0,004	630,00	0,013
716,00	0,004	428,00	0,384
695,00	0,006		
653,00	0,010		
580,00	0,020		
507,00	0,040		
430,00	0,078		
352,00	0,218		
267,00	0,994		
214,00	3,537		

Supplementary Table S2. The following comparison is made of the nuclear magnetic resonance (NMR) values of isolated and purified piscibactin-Fe^{+3} and of the literature piscibactin-Ga^{+3} complexes.

	^{13}C (100 MHz) and ^{1}H NMR (400 MHz) spectra and values for Piscibactin-Fe(III) (D_2O)			^{13}C (125 MHz) and ^{1}H NMR (500 MHz) spectra and values for the Piscibactin-Ga(III) complex (CD_3OD) (Souto et al., 2012)	
**No**	**^{13}C**	**^{1}H**	**COSY**	**^{13}C**	**^{1}H**
**1**	169.2	-	-	164.2	-
**2**	128.8	7.99	8.24-8.15	121.8	6.93, dd (8.5, 1.0)
**3**	128.8	8.10	8.24-8.15	136.4	7.50, ddd (8.5, 7.2, 1.6)
**4**	128.5	7.92	7.92	117.7	6.87, ddd (8.0, 7.2, 1.0)
**5**	129.1	8,54	8.54	132.4	7.58, dd (8.0, 1.6)
**6**	128.7	-		115.3	-
**7**	169.0	-		179.5	-
**8**	22.8	3.91; 3.61		33.8	3.35 dd, (11.2, 1.5)<br>3.73 dd, (11.2, 8.2)
**9**	72.3	4.28		75.3	4.69 ddd (10.0, 8.2, 1.5)
**10**	62.9	4.30		69.1	4.86, d (10.0)
**11**	29.3	3.94; 3.65		37.0	3.07 dd (12.9, 10.7)<br>3.60 dd (12.9, 7.0)
**12**	62.9	4.27		68.1	3.87 dd (10.7, 7.0)
**13**	68.4	4.25		69.2	4.35 dd (5.1, 1.9)
**14**	47.8	3.52, nd		39.2	2.94 dd (17.2, 1.9)<br>3.10 dd (17.2, 5.1)
**15**	169.0	-		184.8	-
**16**	47.6	3.58; 3.54	3.91	39.6	3.40, d (11.6)<br>3.82, d (11.6)
**17**	76.0	-		81.2	-
**18**	12.9	1.28, s		23.4	1.68, s
**19**	179.1	-		179.2	-

Supplementary Table S3. NMR values for the isolated and purified piscibactin and the literature-reported yersiniabactin.

	**^{13}C (100 MHz) and ^{1}H NMR (400 MHz) spectra and values for Piscibactin (D_2O)**			**^{13}C and ^{1}H NMR spectra and values for Yersiniabactin (CD_3OD)** (Chambers et al., 1996)	
**No**	**^{13}C**	**^{1}H**	**COSY**	**^{13}C**	**^{1}H**
1	160.0	-		165.2, s	-
2	128.1	7.30		123.4, d	6.71
3	130.2	7.11		132.7, d	7.26
4	115.2	6.71		118.4, d	6.59
5	129.8	7.01		137.7, d	7.30
6	115.1	-		117. 7, s	-
7	180.3	-		180.8, s	-
8	34.1	2.68; nd	3.81(62.7)	35.6, t	3.24; 3.54
9	62.7	3.81		78.0, d	4.56
10	60.7	4.46		71.1, d	4.71
11	29.0	2.15	4.20 (57.0)	39.4, t	2.98; 3.41
12	57.0	4.20		65.5, t	3.78
13	55.5	4.17		80.4, t	3.44
14	29.2	1.96; 1.30		46.2, s	-
15	180.3	-		193.7, s	-
16	24.6	1.96		40.8, t	3.17; 3.71
17	80.0	-		84.6, s	-
18	18.7	0.93		23.2, q	1.56
19	180.3	-		28.4, q	1.39
20				23.7, q	1.18
21				178.2, s	-
